# A large, multi-center survey assessing health, social support, literacy, and self-management resources in patients with heart failure

**DOI:** 10.1186/s12889-024-18533-7

**Published:** 2024-04-24

**Authors:** Alanna M. Chamberlain, Erinn M. Hade, Irina V. Haller, Benjamin D. Horne, Catherine P. Benziger, Brent C. Lampert, Kismet D. Rasmusson, Kimberly Boddicker, Sheila M. Manemann, Véronique L. Roger

**Affiliations:** 1 Department of Quantitative Health Sciences, Mayo Clinic, Rochester, MN USA; 2 Department of Cardiovascular Medicine, Mayo Clinic, Rochester, MN USA; 3 Department of Population Health, NYU Grossman School of Medicine, New York, NY USA; 4 Essentia Institute of Rural Health, Essentia Health, Duluth, MN USA; 5 Intermountain Medical Center Heart Institute, Salt Lake City, UT USA; 6 Division of Cardiovascular Medicine, Department of Medicine, Stanford University, Stanford, CA USA; 7 Heart and Vascular Center, Essentia Health, Duluth, MN USA; 8 Division of Cardiovascular Medicine, The Ohio State University Wexner Medical Center, Columbus, OH USA; 9 Epidemiology and Community Health Branch, National Heart, Lung, and Blood Institute, National Institutes of Health, Bethesda, MD USA

**Keywords:** Heart failure, Patient-reported information, Survey, Participation bias

## Abstract

**Background:**

Most patients with heart failure (HF) have multimorbidity which may cause difficulties with self-management. Understanding the resources patients draw upon to effectively manage their health is fundamental to designing new practice models to improve outcomes in HF. We describe the rationale, conceptual framework, and implementation of a multi-center survey of HF patients, characterize differences between responders and non-responders, and summarize patient characteristics and responses to the survey constructs among responders.

**Methods:**

This was a multi-center cross-sectional survey study with linked electronic health record (EHR) data. Our survey was guided by the Chronic Care Model to understand the distribution of patient-centric factors, including health literacy, social support, self-management, and functional and mental status in patients with HF. Most questions were from existing validated questionnaires. The survey was administered to HF patients aged ≥ 30 years from 4 health systems in PCORnet® (the National Patient-Centered Clinical Research Network): Essentia Health, Intermountain Health, Mayo Clinic, and The Ohio State University. Each health system mapped their EHR data to a standardized PCORnet Common Data Model, which was used to extract demographic and clinical data on survey responders and non-responders.

**Results:**

Across the 4 sites, 10,662 patients with HF were invited to participate, and 3330 completed the survey (response rate: 31%). Responders were older (74 vs. 71 years; standardized difference (95% CI): 0.18 (0.13, 0.22)), less racially diverse (3% vs. 12% non-White; standardized difference (95% CI): -0.32 (-0.36, -0.28)), and had higher prevalence of many chronic conditions than non-responders, and thus may not be representative of all HF patients. The internal reliability of the validated questionnaires in our survey was good (range of Cronbach’s alpha: 0.50–0.96). Responders reported their health was generally good or fair, they frequently had cardiovascular comorbidities, > 50% had difficulty climbing stairs, and > 10% reported difficulties with bathing, preparing meals, and using transportation. Nearly 80% of patients had family or friends sit with them during a doctor visit, and 54% managed their health by themselves. Patients reported generally low perceived support for self-management related to exercise and diet.

**Conclusions:**

More than half of patients with HF managed their health by themselves. Increased understanding of self-management resources may guide the development of interventions to improve HF outcomes.

**Supplementary Information:**

The online version contains supplementary material available at 10.1186/s12889-024-18533-7.

## Introduction

Heart failure (HF) is a major public health problem, currently affecting more than 6 million Americans and projected to increase to more than 8 million Americans by 2030 [1]. HF is one of the most frequent causes of hospitalizations in the United States [2–4], and is associated with significant mortality, morbidity, and healthcare expenditures, particularly among those aged 65 and older [5]. HF patients are often elderly, and most have multimorbidity [6], which may precipitate acute decompensation and increase the risk of non-fatal complications, healthcare utilization, and death [7–10].

As a chronic disease that coexists with multiple other conditions, HF may cause self-management difficulties in patients who may also have knowledge deficits about HF or a lack of social support to manage their illness [11, 12]. Patients with limited health literacy or social support may have more difficulties accessing healthcare services, interacting with the medical system and communicating with physicians, may less effectively participate in decision-making, and may have more difficulties with self-care including managing medications and adhering to healthy behaviors related to physical activity and diet [11, 13, 14]. Furthermore, self-care behaviors, including managing medications, adherence to a healthy diet and physical activity, and monitoring for changes in symptoms may need to be adapted over time as HF progresses, which may hinder effective self-care [15].

Understanding the social and self-management resources that patients draw upon to effectively manage their health is fundamental to design new practice models to improve outcomes in HF. Patients with HF who have more effective self-care behavior have improved quality of life and lower rates of HF and all-cause hospital readmissions [16–18], yet randomized controlled trials targeting dietary and exercise interventions have exhibited no to modest improvements in outcomes in patients with HF [19, 20]. Thus, we implemented a survey guided by the Chronic Care Model [21] to understand the distribution of patient-centric factors, including health literacy, social support, and self-management, as well as self-reported functional and mental status in patients with HF. We selected existing validated questionnaires measuring social support, health literacy, and self-management because these are conceptually essential to successfully manage HF.

The current study was designed to comprehensively assess barriers to successful self-management in a real-world population of patients with HF from multiple health care systems with linkage to data from the electronic health record to inform the design of future interventions for improvement of patient outcomes. The survey was administered to HF patients from 4 health care systems in the United States participating in a Patient-Centered Outcomes Research Institute (PCORI) clinical data research network. The purpose of this article is to describe the rationale and conceptual framework of the survey, the constructs included in the survey, the implementation of the survey at the 4 participating sites, and the linkage of clinical data to survey responses. Herein we describe the response rate for our survey and reasons for non-response, characterize differences between survey responders and non-responders, and summarize patient characteristics and responses to the survey constructs among responders.

## Methods

### Patient-Eentered Eetwork of Eearning Eealth Eystems

The Patient-Centered Network of Learning Health Systems (LHSNet) was a clinical data research network of 9 participating organizations, including 6 health systems, encompassing data on nearly 10 million patients [22]. LHSNet was funded by PCORI from 2015 to 2019. More details on the LHSNet clinical data research network partners and organization structure has been previously published [22]. Briefly, each of the participating sites mapped their data to a standardized National Patient-Centered Clinical Research Network (PCORnet) Common Data Model, allowing efficient execution of data queries across LHSNet and PCORnet [23–25]. The LHSNet developed computable phenotypes for a common disease (HF) [26] and a rare disease (osteogenesis imperfecta), and designed surveys for these 2 patient cohorts, along with a weight cohort (overweight and obesity) [27] based on the PCORnet obesity algorithm. Four health systems from LHSNet – Essentia Health, Intermountain Health, Mayo Clinic, and The Ohio State University – participated in the HF survey.

### Identification of patients with heart failure

A series of computable phenotypes for HF were developed utilizing data elements available in the PCORnet Common Data Model [26]. The computable phenotypes varied in complexity, ranging from a single diagnosis code to inclusion of diagnosis code(s) plus prescription medications and NT-proBNP. For this study, we chose a computable phenotype that had higher positive predictive value to reduce false positive diagnoses. HF was identified in patients aged 30 years or older using the algorithm that required 2 or more HF diagnostic codes (*International Classification of Diseases, Ninth Revision* (ICD-9) 428.xx and ICD-10 I50.xx) and 1 or more HF-related prescription drugs (aldosterone antagonists; HF specific beta blockers including bisoprolol, carvedilol, and metoprolol succinate; loop diuretics; digoxin; angiotensin converting enzyme inhibitors; angiotensin receptor blockers; sucabatril/valsartan; ivabradine; and hydralazine/isosorbide dinitrate in Black patients). We required the 2 or more HF diagnostic codes to occur more than 30 days apart and within a 2 year period, and the HF-related prescription to occur within the same 2 year period. The performance of this algorithm was tested against a Framingham criteria-validated cohort of patients with HF in Olmsted County, MN and found to have a sensitivity of 56.1%, specificity of 99.6%, positive predictive value of 80.7%, and negative predictive value of 98.7% [26]. We restricted our sampling frame for the HF survey to patients with recent diagnoses of HF (first ever diagnosis of HF on or after 1/1/2013 at 1 of the participating sites and on or after 1/1/2015 for the other 3 participating sites).

### Selection of survey questions

We used the Chronic Care Model as a conceptual model to guide the selection of questions for our survey. The Chronic Care Model is one approach to improving chronic illness care and proposes to change reactive acute-oriented care to care that is proactive and patient-centered. The Chronic Care Model elucidates the requisite elements for improving chronic illness care, including health systems and community requirements, specifically highlighting the importance of self-management support [21, 28, 29]. Both health literacy and social support affect how patients interact with the medical system, participate in decision-making, and practice self-care behaviors [11, 13, 14], and are thus instrumental in improving self-management. Effective self-management has been associated with improved health outcomes in chronic diseases, such as asthma, hypertension, and diabetes [30, 31]. These key patient-centric factors, social support, health literacy, and self-management are conceptually essential to successfully manage HF. A prior meta-analysis reported reduced combined endpoint of HF-related hospitalization or all-cause death, reduced HF-related hospitalization alone, and modestly improved HF-related quality of life for HF patients receiving self-management interventions [18].

Most questions in the survey were from existing validated questionnaires. Social support was measured by the Patient-Reported Outcomes Measurement Information System (PROMIS) surveys measuring components of instrumental support, informational support, and social isolation [32]. In addition, 2 questions from the National Health and Aging Trends Study were included, which assessed whether a patient has someone accompany them to doctor visits and helps with health care activities [33, 34]. Health literacy was assessed using the Health Literacy Screener [35–37]. Patient self-management was assessed by the Chronic Illness Resources Survey [38, 39], which includes 7 subscales assessing support from proximal (family and friends) to distal (neighborhood or community) factors. To ensure we obtained measures of functional and mental status at the time of the survey, we also included a question related to general health [40], a question asking participants to self-report cardiovascular related diseases, a question about activities of daily living (ADLs) and mobility activities, and the PROMIS Health Profile [41, 42], which includes 29 questions related to various aspects of mental and physical health. In total, the survey included 85 questions, which are summarized in Table 1.

**Table 1:** Components of the heart failure survey

Construct	Measure	Brief description	Reliability	Items
Global Health	General health question [40]	Assesses overall general health rated as: Excellent, Very Good, Good, Fair, Poor.		1
	Self-report disease status	Assesses which cardiovascular related diseases that the patient self-reports.		1
Functional and Mental Status	PROMIS 29 v2.0-Health Profile [41, 42]	Assesses health and well-being (includes Anxiety, Depression, Fatigue, Pain, Physical Function, Sleep Disturbance, and Ability to Participate in Social Roles and Activities subscales).	Cronbach’s alpha for subscales: 0.85–0.96	29
	Basic and instrumental activities of daily living and mobility activities	Assesses functional disability.		9
Self-Management	Chronic Illness Resources Survey [38, 39]	Assesses perceived support and community resources for self-management (includes Physician/Health Care Team, Family and Friends, Dietary, Exercise, Personal, Neighborhood/Community, Work, Media and Policy, and Organizations subscales).	Cronbach’s alpha for subscales: 0.50–0.84	22
Social Support	PROMIS SF v2.0– Informational Support [32]	Assesses perceived availability of information or advice.	Cronbach’s alpha = 0.94	4
	PROMIS SF v2.0– Instrumental Support [32]	Assesses perceived availability of support for cognitive, material, or task performance.	Cronbach’s alpha = 0.91	4
	PROMIS SF v2.0—Social Isolation [32]	Assesses perceptions of social isolation.	Cronbach’s alpha = 0.91	4
	Accompaniment to physician visits [33, 34]	Assesses whether patient has someone accompany them to doctor visits.		1
	Handling of health care activities [33, 34]	Assesses whether patient has someone help them with health care activities.		1
Health Literacy	Health Literacy Screener [35–37]	Assesses difficulty understanding information or performing reading tasks in health care setting.	Cronbach’s alpha = 0.80	3
Demographics	Race, ethnicity, marital status, education	Assesses general demographic information.		6

### Survey implementation

The Mayo Clinic Survey Research Center designed and printed the surveys for all participating sites. The survey was printed in English. A consecutive sample of the first 1000 eligible patients were selected from Intermountain Health, whereas all eligible patients were invited for participation at the other 3 sites. Intermountain Health was responsible for printing and assembling the rest of the survey packet and mailing of the surveys for their patients. For Essentia Health and The Ohio State University, patients who met the eligibility criteria for participation were first sent an introductory letter from the site principal investigator. This introductory letter served to inform the patient of the study, the nature of the collaboration between their site and Mayo Clinic, and allowed the patient the opportunity to opt out of the study (via telephone call to the site principal investigator or by checking a box on the introductory letter and returning it via mail) before being mailed a survey. The patients were given 3 weeks after the introductory letter mailing to opt out; for those who did not opt out, the names and addresses of patients were forwarded to the Mayo Clinic Survey Research Center using a secure file transfer protocol. The Mayo Clinic Survey Research Center assembled the survey packets and mailed the surveys for Mayo Clinic, Essentia Health, and The Ohio State University.

The survey packet included a cover letter, Health Insurance Portability and Accountability Act of 1996 (HIPAA) authorization form, survey, and postage paid and addressed return envelope. In accordance with ethical guidelines, the cover letter included written statements that patient participation in the study will not impact their health care at the participating site and that they could refuse study participation without any negative consequences. In addition, if the patient did not wish to participate, they were allowed the opportunity to check a box on the cover letter indicating ‘I am not willing to participate in this research study’ and return the letter in the postage paid envelope. For all sites, a second mailing of the survey packet was sent to non-responders approximately 4 weeks after the initial mailing. For Mayo Clinic only, telephone contact was attempted approximately 4 weeks after the second mailing; patients were given the opportunity to complete the survey over the phone or were mailed another survey if requested. When completed surveys were returned without an accompanying signed HIPAA form, an additional mailing of the HIPAA form was attempted. If a signed HIPAA form was not obtained, the survey was not used. The surveys were completed between 10/7/2014 and 11/14/2018. The completed surveys for all 4 sites were sent for scanning at the Mayo Clinic Survey Research Center, who returned a survey dataset to the site principal investigator. A SAS query was written and distributed to all sites for scoring of the survey responses.

### Clinical data collection

The majority of the remaining data elements, including demographic information and comorbidities, were available from the PCORnet Common Data Model. A SAS query was written and distributed to all sites for collection of demographics and comorbidities present at the survey date (cross-sectional study) to ensure a standardized approach was used at all sites for defining these variables. All height and weight values within the 5 years prior to survey date were obtained and averaged. The most recent values of height and weight were used to calculate body mass index (BMI) as weight (in kg) divided by height (in meters) squared, as long as the values were not ≥ 20% lower or higher than the mean values. If a height and/or weight were ≥ 20% lower or higher than the mean, the value was excluded and the next proximal value that was not ≥ 20% different from the mean was selected. Current, former, and never smoking status were obtained at the date of the survey. Comorbidities were ascertained using the United States Department of Health and Human Services list of 20 chronic conditions for studying multimorbidity [43, 44]. We excluded autism and human immunodeficiency virus due to low prevalence. In addition, we added anxiety to the list of chronic conditions because it is common in patients with HF. For each chronic condition, we required 2 occurrences of a code (either the same diagnostic code or 2 different diagnostic codes within the same code set) separated by more than 30 days and occurring within the 5 years prior to the survey date to rule out false positives due to suspect diagnoses. The ICD-9 and ICD-10 codes used to define the conditions are provided in Additional File 1. Left ventricular ejection fraction was not available in the Common Data Model and was pulled from local electronic medical records. The closest ejection fraction within the year prior to the survey date was obtained. Finally, residence was defined based on the zip code using the primary Rural-Urban Commuting Area (RUCA) classification as metropolitan/urban, micropolitan/large rural, small town/small rural, and rural/isolated rural [45].

### Statistical analysis

Analyses were performed using SAS statistical software, version 9.4 (SAS Institute Inc., Cary, NC). The response rate was calculated using the American Association for Public Opinion Research (AAPOR) formula 2, as the number of complete and partial surveys divided by the total number of surveys including complete and partial surveys, refusals, non-contacts, others, and cases of unknown eligibility [47]. Internal reliability of each of the validated questionnaires included in our survey was assessed with Cronbach’s alpha using the combined data from all 4 sites. Characteristics of survey responders vs. non-responders were compared using standardized differences, calculated as the difference in means or proportions divided by the standard error; confidence intervals were calculated using methodology by Hedges and Olkin [46]. For the 2 sites utilizing the introductory letter allowing patients to opt out of the study, data were not included for patients who opted out. In addition, data were not available in the PCORnet Common Data Model for some nonresponders at 2 sites (81 at one site and 48 at another site) so their data could not be included in the comparison of responders and non-responders. Among survey responders, summaries of the survey constructs were provided using percentages for categorical variables, and median (25th, 75th percentile) and range for continuous variables. Patients who returned partially completed surveys were retained for analysis; however, missing data was not imputed. For the patients with partial surveys, data was only included for the subscales where all questions were answered and a score could be estimated.

## Results

**Fig. 1:**
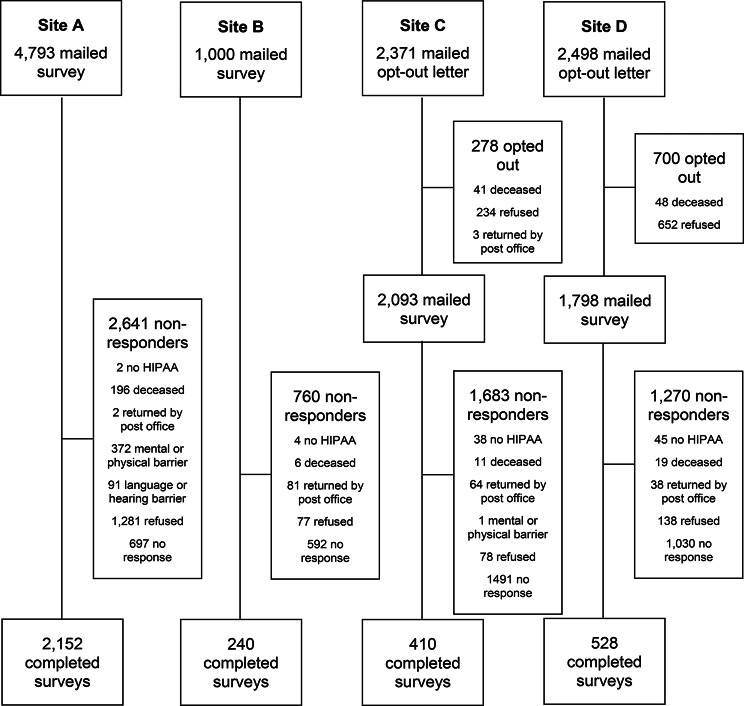
Flow chart

Across the 4 participating sites, 10,662 patients with HF were identified and invited to participate in the survey (Fig. 1). The response rate varied across sites (17.3-44.9%), with an overall response rate of 31.2% (3330 returned surveys). 60% of respondents (1992) returned complete surveys, whereas the remaining returned partial surveys with at least one missing question. Among the 7332 non-responders, the most common reasons for nonparticipants included: no response (3810; 52.0%), refusal (2460; 33.6%), and mental, physical, language, or hearing barrier (464; 6.3%). In addition, 89 patients returned the survey without a signed HIPAA authorization form, and thus the survey could not be used. Responders were older (73.5 vs. 71.2 years), less racially diverse (3.2% vs. 11.5% non-White), less likely to be a current smoker (8.8% vs. 17.5%), less likely to have reduced ejection fraction (EF; 21.7% vs. 27.3% with EF < 40%), and had higher prevalence of many chronic conditions than non-responders (Table 2).

**Table 2:** Characteristics of survey responders compared to non-responders

	Responders (*N* = 3330)	Non-responders (*N* = 6225)	Standardized difference (CI)
Site			
A	2152 (64.6)	2641 (42.4)	0.457 (0.414, 0.499)
B	240 (7.2)	679 (10.9)^*^	-0.129 (-0.171, -0.087)
C	410 (12.3)	1683 (27.0)^†^	-0.377 (-0.419, -0.334)
D	528 (15.9)	1222 (19.6)^§^	-0.099 (-0.141, -0.057)
Age, years	73.5 (12.0)	71.2 (14.8)	0.177 (0.134, 0.219)
Unknown, n	7	1	
Male sex	1845 (55.6)	3355 (54.1)	0.030 (-0.012, 0.072)
Unknown, n	9	19	
Non-white race	106 (3.2)	699 (11.5)	-0.320 (-0.363, -0.277)
Unknown, n	44	130	
Hispanic ethnicity	34 (1.0)	139 (2.3)	-0.097 (-0.139, -0.055)
Unknown, n	34	83	
Residence			
Metropolitan/urban	1628 (48.9)	3171 (51.0)	-0.042 (-0.084, 0.000)
Micropolitan/large rural	1079 (32.4)	1811 (29.1)	0.071 (0.029, 0.114)
Small town/small rural	305 (9.2)	595 (9.6)	-0.014 (-0.056, 0.028)
Rural/isolated rural	317 (9.5)	642 (10.3)	-0.027 (-0.069, 0.015)
Unknown, n	1	6	
Body mass index, kg/m^2^			
<25	443 (15.3)	978 (19.6)	-0.115 (-0.161, -0.069)
25 to < 30	837 (28.8)	1372 (27.5)	0.029 (-0.017, 0.075)
≥30	1625 (55.9)	2637 (52.9)	0.062 (0.016, 0.107)
Unknown, n	425	1238	
Smoking status			
Current	230 (8.8)	855 (17.5)	-0.262 (-0.309, -0.214)
Former	1105 (42.1)	2082 (42.7)	-0.012 (-0.059, 0.036)
Never	1288 (49.1)	1928 (39.8)	0.189 (0.141, 0.237)
Unknown, n	707	1350	
Ejection fraction, %			
<40	441 (21.7)	823 (27.3)	-0.129 (-0.186, -0.073)
40–49	407 (20.0)	494 (16.4)	0.095 (0.039, 0.151)
≥50	1183 (58.3)	1701 (56.4)	0.038 (-0.018, 0.094)
Unknown, n	1299	3207	
Hypertension	2354 (70.7)	3542 (56.9)	0.290 (0.247, 0.332)
Coronary artery disease	1537 (46.2)	2090 (33.6)	0.259 (0.217, 0.301)
Cardiac arrhythmias	2063 (62.0)	2816 (45.2)	0.340 (0.298, 0.382)
Hyperlipidemia	2043 (61.4)	2799 (45.0)	0.333 (0.291, 0.375)
Stroke	354 (10.6)	540 (8.7)	0.066 (0.024, 0.108)
Arthritis	1097 (32.9)	1458 (23.4)	0.213 (0.171, 0.255)
Asthma	265 (8.0)	362 (5.8)	0.085 (0.043, 0.127)
Cancer	802 (24.1)	936 (15.0)	0.230 (0.187, 0.272)
Chronic kidney disease	832 (25.0)	1372 (22.0)	0.070 (0.027, 0.112)
Chronic obstructive pulmonary disease	508 (15.3)	773 (12.4)	0.082 (0.040, 0.124)
Dementia	79 (2.4)	392 (6.3)	-0.194 (-0.236, -0.151)
Depression	462 (13.9)	761 (12.2)	0.050 (0.007, 0.091)
Diabetes	1131 (34.0)	1538 (24.7)	0.204 (0.162, 0.247)
Hepatitis	27 (0.8)	63 (1.0)	-0.021 (-0.063, 0.021)
Osteoporosis	227 (6.8)	357 (5.7)	0.045 (0.003, 0.087)
Schizophrenia	18 (0.5)	74 (1.2)	-0.070 (-0.112, -0.028)
Substance abuse disorders	151 (4.5)	349 (5.6)	-0.049 (-0.091, -0.007)
Anxiety	272 (8.2)	523 (8.4)	-0.009 (-0.051, 0.034)

Values are reported as N (%) or mean (SD)

^*^Excludes 81 non-responders whose data were no longer available in the Common \Data Model at the time of the data pull

^†^Excludes 278 patients who opted out to the initial contact letter

^§^Excludes 700 patients who opted out to the initial contact letter, and an additional 48 non-responders whose data were no longer available in the Common Data Model at the time of the data pull

The internal reliability of the validated questionnaires included in our survey was generally good (range of Cronbach’s alpha: 0.50–0.96; Table 1). The majority of responders filled out the survey themselves without assistance (87.6%; Table 3). More than half (58.2%) were married and 22.9% were widowed. Most patients described their health as generally good (42.9%) or fair (33.8%; Table 4). Responders frequently had cardiovascular comorbidities, and more than half had difficulty climbing stairs. In addition, more than 10% of responders reported difficulties with bathing, preparing meals, and using transportation, and 29% of responders reported difficulty with at least 1 activity of daily living (out of a list of 8 activities). Although nearly 80% of patients had family or friends sit with them during a doctor visit, more than half (53.7%) responded that they manage their health mostly by themselves. Patients generally had high health literacy and high levels of both instrumental support (perceived availability of information or advice) and informational support (perceived availability of support for cognitive, material, or task performance; Table 5). In addition, the greatest perceived support/resources for self-management was related to health care (shared decision making, active listening, and efforts to ensure understanding). Participants reported lower perceived support for self-management from community organizations, exercise, diet, and family/friends (which includes questions related to exercising and sharing healthy recipes with friends or family, or having family/friends prepare healthy food for you).

**Table 3:** Summary of demographics for respondents

	N Missing	N (%) among responders
Marital status	47	
Married		1909 (58.2)
Living with someone in a marriage-like relationship		72 (2.2)
Divorced		341 (10.4)
Separated		31 (0.9)
Widowed		751 (22.9)
Never been married		179 (5.5)
Education	61	
≤8th grade		107 (3.3)
Some high school		202 (6.2)
High school graduate or GED		1071 (32.8)
Vocational, technical, or business school		413 (12.6)
Some college or Associates degree		659 (20.2)
Bachelor’s degree (4-year college graduate)		395 (12.1)
Graduate or professional school		388 (11.9)
Other		34 (1.0)
Employment status	63	
Working full time (≥ 35 h per week)		340 (10.4)
Working part time		201 (6.2)
Not currently working for pay		2726 (83.4)
Reason not currently working for pay	24	
Seasonal worker		6 (0.2)
Homemaker		107 (4.0)
In school		2 (0.1)
Retired		2273 (84.1)
Disabled		434 (16.1)
Other		79 (2.9)
Person who filled out the survey	54	
Patient without assistance		2868 (87.6)
Patient with assistance		228 (7.0)
Spouse		95 (2.9)
Family member		73 (2.2)
Caregiver or health care provider		8 (0.2)
Other		4 (0.1)

**Table 4:** Summary of general health, chronic conditions, functional limitations, and handling of health care for respondents

	N Missing	N (%) among responders
‘In general, would you say your health is…’	112	
Excellent		54 (1.7)
Very good		452 (14.1)
Good		1380 (42.9)
Fair		1088 (33.8)
Poor		244 (7.6)
‘Do you currently have, or in the past have you experienced…’	0	
High blood pressure		2402 (72.1)
High cholesterol		1712 (51.4)
Diabetes		1069 (32.1)
Atrial fibrillation		1359 (40.8)
Heart attack		880 (26.4)
Stroke		380 (11.4)
Some or much difficulty or unable to do activities of daily living		
Bathing	58	541 (16.5)
Getting in and out of bed	68	287 (8.8)
Feeding yourself	48	59 (1.8)
Dressing	53	238 (7.3)
Using the toilet	54	147 (4.5)
Preparing meals	207	425 (13.6)
Managing medications	123	292 (9.1)
Using transportation	92	497 (15.4)
Number of activities of daily living reported with some or much difficulty or unable to do	0^*^	
0		2365 (71.0)
1		396 (11.9)
≥2		569 (17.1)
Some or much difficulty or unable to do mobility activities		
Climbing 2 flights of stairs without stopping to rest	83	1785 (55.0)
‘In the last year, did anyone (family, friend) sit in with you and your doctor during visits?’	66	
Yes		2591 (79.4)
No		673 (20.6)
‘People today are asked by their doctors and other health care providers to do many things to stay healthy or treat health problems…. How do you usually handle these things?’^†^	54	
Mostly by myself		1760 (53.7)
Together with family or close friends		1135 (34.7)
Family or close friends mostly handle		162 (5.0)
It varies		219 (6.7)

^*^Persons missing answers to individual activities were assumed to not have difficulty with that activity when counting the number of activities of daily living reported with difficulty/unable to do

^†^The full question states, ‘People today are asked by their doctors and other health care providers to do many things to stay healthy or treat health problems — for example, manage medicines, get tests and lab work done, watch weight and blood pressure, or have yearly exams. How do you usually handle these things?’

**Table 5:** Summary of functional and mental status, social support, health literacy, and self-management for respondents

	N Missing	Mean (SD)	Median (25th, 75th percentile)	Possible range of scores^*^
PROMIS 29 Health Profile subscales^†^				
Physical function	150	10.2 (5.2)	9 (5, 14)	4–20
Social roles	167	9.9 (4.7)	10 (5, 13)	4–20
Anxiety	97	6.4 (3.2)	5 (4, 8)	4–20
Depression	138	6.3 (3.3)	4 (4, 8)	4–20
Fatigue	156	10.0 (4.1)	9 (7, 13)	4–20
Sleep disturbance	172	9.6 (4.1)	9 (7, 12)	4–20
Pain	185	8.8 (8.4)	8 (4, 12)	4–20
‘In the past 7 days, how would you rate your pain on average?’^†^	130	3.0 (2.6)	3 (1, 5)	0–10
PROMIS Instrumental Support^§^	177	17.0 (6.1)	19 (15, 20)	4–20
PROMIS Informational Support^§^	120	16.7 (6.7)	17 (15, 20)	4–20
PROMIS Social Isolation^†^	136	7.1 (3.6)	6 (4, 9)	4–20
Health Literacy^§^	95	12.0 (3.0)	13 (10, 15)	3–15
Chronic Illness Resources Survey subscales^§^				
Physician/health care team	191	12.0 (5.9)	12 (10, 14)	3–15
Family and friends	151	6.0 (5.8)	5 (4, 7)	3–15
Personal	221	9.2 (6.0)	9 (7, 12)	3–15
Neighborhood/community	161	7.9 (5.9)	7 (6, 9)	4–20
Media and policy	142	9.6 (2.9)	10 (8, 11)	3–15
Organizations	107	4.7 (2.8)	4 (3, 6)	3–15
Work	0^**^	8.9 (3.9)	9 (6, 12)	3–15
Dietary	144	6.5 (5.7)	6 (5, 8)	3–15
Exercise	142	5.7 (5.8)	5 (3, 7)	3–15

^*^Presents raw scores. Range of possible scores is based on the full range of survey response options (not the range of scores in our cohort)

^†^Higher scores indicate worse health or more social isolation

^§^Higher scores indicate better social support, higher health literacy, or higher self-management resources

^**^Patients were instructed to leave the work questions blank if they did not work. The mean and median values are based on 634 respondents who filled out the work questions

## Discussion

We implemented a large, survey of patients with HF seen at 4 health care systems participating in PCORnet® and linked the survey responses with data from the electronic health record. The survey was informed by the Chronic Care Model and included standardized questionnaires measuring constructs related to functional status, mental health, health literacy, social support, and self-management resources. The response rate varied across sites, but was overall 31%. Responders tended to be older and have more chronic conditions than non-responders. In addition, it is noteworthy that the survey respondents were primarily of White race (97%) and had generally high health literacy; thus, our findings should be interpreted in this context. Nevertheless, inclusion of patients with HF seen at 4 health systems may have improved the generalizability of our results over a single center survey.

Patients with HF have many comorbidities which often complicate their management, and the complexity of treatment plans for these patients may encumber self-management. Self-management encompasses three sets of tasks, including medical management of the condition; maintaining, changing, and creating new behaviors; and coping with emotions commonly experienced by having a chronic condition [48]. In HF, self-management behaviors include activities such as smoking cessation, adhering to a healthy diet, exercising, and taking medication (self-care maintenance); daily weighing, monitoring blood pressure, and observing changes in fatigue, shortness of breath, and activity level (self-care monitoring); and adjusting medications such as diuretics, adapting activity level and diet, and consulting a health care professional in response to changes in symptoms when they occur (self-care management) [15].

In the current study, most patients with HF had high health literacy, as well as informational and instrumental support. However, patients reported generally low perceived support for self-management related to self-care maintenance practices of exercise and diet, important modifiable risk factors in the long-term management of HF. Recent HF guidelines from the American Heart Association/American College of Cardiology/Heart Failure Society of America with class I level of evidence say we should provide multidisciplinary education and support to promote self-care in patients with HF and to reduce potential medical and social barriers to self-care [49]. Recommendations from the Heart Failure Association of the European Society of Cardiology outlines lifestyle and behavior recommendations, which include diet and physical activity, to promote self-care maintenance and management in patients with HF [15]. Evidence from systematic reviews and meta-analyses shows that patients with HF who have more effective self-care behavior have lower rates of HF and all-cause hospital readmissions and modestly improved quality of life [16–18]. Furthermore, large multi-center randomized controlled trials showed modest effects of exercise training on the composite endpoints of all-cause mortality or hospitalization and cardiovascular mortality or HF hospitalization [20], but no effect of a dietary intervention to reduce sodium intake on outcomes in patients with HF [19]. Thus, increased understanding of self-management resources for lifestyle behaviors may be needed to design interventions to improve outcomes in patients with HF.

### Future directions

Although the data in the current study are primarily cross-sectional, due to the availability of patient identifiers at each of the sites, it would be possible to pull in additional data from the electronic health record to obtain follow-up information and relevant patient outcomes for future studies. Furthermore, linkage to other data sources could bring in additional data that may not be available in the medical records, such as publicly available data on neighborhood characteristics from the Census or American Community Survey, measures of neighborhood socioeconomic status, or information on causes of death from the National Death Index. In addition, the available data on survey non-responders would allow further research to understand participation bias and to inform future design and implementation of surveys to improve the generalizability of survey findings. Finally, a unique aspect to our survey implementation was the utilization of a single survey research center for survey administration at 3 of the 4 sites. Our process can serve as a guide for future multi-center surveys, but additional research may be warranted to identify methods to improve inclusion of sites who may lack resources to facilitate survey administration and to streamline processes for multi-center surveys utilizing a single survey research center.

### Limitations and strengths

Some limitations deserve mention. First, we relied on a computable phenotype to identify patients with HF using electronic health record data, so some misclassification in the diagnosis of HF may have occurred. Second, our survey was long, including 85 questions, which may have affected our response rate (31%) and may have contributed to return of partial surveys with at least one missing question (40% of respondents). Third, we observed some notable differences in characteristics of responders vs. non-responders. Fourth, the vast majority of respondents were non-Hispanic Whites. Taken collectively, these may have affected the external validity of our results. Thus, the results of our study should be interpreted in this context because our findings may not be generalizable to all patients with HF. Fifth, we did not include questions in the survey related to medical management of HF or treatment burden in general, which may influence self-management. Nevertheless, we leveraged the PCORnet infrastructure to efficiently identify a cohort of HF patients with similar inclusion criteria across 4 health systems, and implemented a large survey to gather critical information not included in electronic medical records, and linked our survey data with data from the electronic health record.

## Conclusions

We implemented a survey guided by the Chronic Care Model to understand the distribution of patient-centric factors, including health literacy, social support, self-management, and functional and mental status in patients with HF. Most patients with HF in this study described their health as generally good or fair, had high health literacy and social support, and most frequently endorsed health care as the greatest perceived support/resource for self-management. However, patients reported generally low perceived support for self-management related to exercise and diet, indicating that knowledge and social support may not be sufficient for effective self-management. Furthermore, more than half of patients with HF manage their health by themselves. Increased understanding of self-management resources for lifestyle behaviors such as diet and exercise, in particular, may guide the development of interventions to improve HF outcomes.

### Electronic supplementary material

Below is the link to the electronic supplementary material.

Supplementary Material 1

## Data Availability

The participants of this study did not provide written informed consent for storage of their data on an individual level in repositories or journals. However, requests to access the data set from qualified researchers trained in human subject confidentiality protocols will be considered. Access to the data will require approval of the proposed use of the data and establishment of data use agreements with each of the participating health systems. Researchers who wish to access the data set should send a request to the corresponding author.

## Abbreviations

AAPOR: American Association for Public Opinion Research
ADLs: activities of daily living
BMI: body mass index
HIPAA: Health Insurance Portability and Accountability Act of 1996
HF: heart failure
LHSNet: Patient-Centered Network of Learning Health Systems
PCORI: Patient-Centered Outcomes Research Institute
PCORnet: The National Patient-Centered Clinical Research Network
PROMIS: Patient-Reported Outcomes Measurement Information System
RUCA: Rural-Urban Commuting Area
